# A Comparative Analysis of Transcriptome-Wide Differential Gene Expression and Alternative Polyadenylation in the Ovaries of Meat Ducks and Laying Ducks

**DOI:** 10.3390/ani16020313

**Published:** 2026-01-20

**Authors:** Sike Wang, Yaomei Wang, Shiwei Li, Chao Jia, Debing Yu, Weiling Huang

**Affiliations:** 1 College of Animal Science, Xizang Agricultural and Animal Husbandry University, Linzhi 860000, China; 2 Department of Animal Genetics, Breeding and Reproduction, College of Animal Science and Technology, Nanjing Agricultural University, No. 1 Weigang, Nanjing 210095, China

**Keywords:** duck, ovary, full-length transcriptome, alternative polyadenylation (APA), reproductive performance

## Abstract

Significant differences in reproductive performance exist between meat-type ducks (e.g., Qiangying Duck, QD) and laying-type ducks (e.g., Shaoxing Duck, SD). The molecular mechanisms underlying these differences, particularly concerning ovarian development and function, remain incompletely understood. This study aimed to comprehensively characterize the ovarian transcriptomes of these two duck types, focusing on differential gene expression and post-transcriptional regulatory events. We performed an integrated full-length transcriptome analysis of ovarian tissues from these two breeds using PacBio SMRT and Illumina sequencing. Bioinformatic analyses, including functional annotation, differential expression analysis, and the identification of APA events, were used. We discovered substantial breed-specific differences in alternative polyadenylation (APA), with SD ducks exhibiting significant 3′UTR shortening in 3799 genes and 3′UTR lengthening in 1626 genes compared to QD. The integrated analysis of differential gene expression and APA events highlighted key genes related to steroid hormone synthesis (*HMGCS1*, *DHCR24*), lipid metabolism (*SCD*), signal transduction (*HRAS*), and antioxidant defense (*SOD1*). The functional enrichment implicated critical pathways such as mitochondrial energy metabolism, oxidative phosphorylation, and fatty acid degradation. Our study provides a comprehensive atlas of post-transcriptional regulation in the duck ovary and reveals APA as a crucial process of gene regulation. APA may contribute to the differential ovarian function and egg-laying capacity between meat and laying ducks, thus offering valuable targets for genetic selection.

## Simple Summary

We aimed to understand why some duck breeds excel in egg production while others grow faster but lay fewer. Their ovaries are the key organs related to egg-laying, so we compared the complete set of active genes in the ovaries between the high-yielding Shaoxing ducks and the low-yielding Qiangying ducks. Using advanced full-length transcriptome sequencing, we discovered nearly a hundred thousand novel gene fragments and divergent patterns of alternative polyadenylation (APA), a key post-transcriptional regulatory process, in the two breeds. These differences affect energy use, hormone production, and cell protection. By linking these gene-level changes to ovarian function and breed phenotype, our work identifies candidate genes and regulatory mechanisms that may contribute to divergent egg-laying performance. These findings provide foundational insights and testable hypotheses for future breeding strategies aimed at enhancing lay efficiency in ducks.

## 1. Introduction

Ducks in the waterfowl industry are primarily categorized into meat-type and egg-type breeds based on their economic purpose. Meat-type ducks, such as the Qiangying duck (QD), are selected for rapid growth and superior meat quality. In contrast, laying-type ducks, like the Shaoxing duck (SD), are renowned for their high egg production and consistent reproductive performance. Research has traditionally focused on the distinct strengths of each type: on growth and meat quality genetics (e.g., *MYOD1* [1], *MYF5* [2]) in meat-type ducks and on hormonal regulation of egg production and differential expression of reproduction-related genes in laying ducks [3,4]. This dichotomy has left a significant knowledge gap regarding the fundamental molecular mechanisms responsible for their differences in reproductive performance, particularly the systematic regulation of ovarian development and function.

The ovary is the core organ of the female reproductive system, responsible for oogenesis, steroid hormone secretion, and regulating the reproductive cycle [5,6]. Its development, from embryogenesis through folliculogenesis [7], is fundamental to laying performance. This indicates potential divergence in ovarian development and response to hormonal signals between the breeds, yet the molecular basis of which remains elusive.

**Figure 1:**
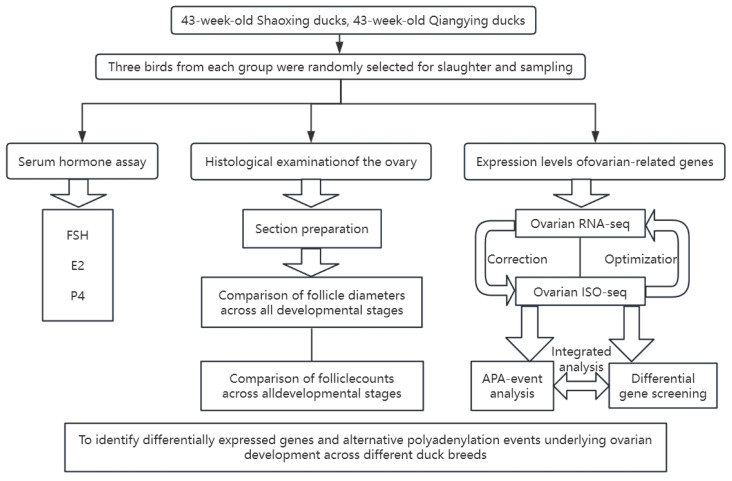
A schematic overview of the study design. Abbreviations in the figure: FSH: Follicle-Stimulating Hormone; E2: Estradiol; P4: Progesterone; RNA-seq: RNA sequencing; ISO-seq: Isotope-labeled sequencing (or Isoform sequencing, depending on context); APA: Alternative Polyadenylation.

While next-generation sequencing (NGS) platforms such as Illumina are powerful for quantifying gene expression, their inherent short read lengths limit the accurate reconstruction of full-length transcripts and impede the detection of complex post-transcriptional modifications [8]. Third-generation sequencing technologies, such as PacBio Single Molecule Real-Time (SMRT) sequencing, overcome this limitation by generating long reads that can easily span entire transcripts from the 5′ cap to the polyA tail without assembly [9,10]. This allows for precise identification of transcript isoforms, alternative splicing (AS), alternative polyadenylation (APA), and other structural variations [11]. We hypothesized that divergent reproductive performance between meat-type and laying-type ducks stems from distinct transcriptional and post-transcriptional regulatory programs in the ovary. Specifically, we postulated that breed-specific differences in gene expression, AS, and APA collectively dysregulate key biological pathways critical for ovarian function. To test this, we performed an integrated analysis of the ovarian transcriptomes of QD and SD ducks using both PacBio SMRT and Illumina sequencing platforms. The objectives were to (1) construct a comprehensive full-length transcriptome map of the duck ovary, (2) identify differentially expressed genes (DEGs) between the two breeds, (3) characterize global patterns of AS and APA, and (4) integrate these data to elucidate potential molecular mechanisms underlying their differential ovarian function and egg-laying capacity. Particularly, we focused on APA, as it is known to regulate gene expression by altering mRNA stability, translation efficiency, and localization. These processes could be highly relevant to the dynamic steroidogenic and metabolic environment of the ovary. A schematic overview of the study design is provided in Figure 1.

## 2. Materials and Methods

### 2.1. Ethics Statement

All experimental procedures involving ducks were reviewed and approved by the Institutional Animal Care and Use Committee (IACUC) of Xizang Agricultural and Animal Husbandry University (Approval Protocol Number: [XZA-2025-36]). The study was conducted in strict accordance with the institutional guidelines, the National Guidelines for the care and use of Laboratory Animals in China, and internationally recognized principles. Every effort was made to minimize animal numbers and suffering.

### 2.2. Animals and Samples

A total of six 43-week-old female ducks were used in this study: three Shaoxing Ducks (SD) and three Qiangying Ducks (QD). The two breeds represent distinct production types: QD is a meat-type breed selected for rapid growth, with an average annual egg production of approximately 247–250 eggs (DB34/T 4651-2023) [12]. In contrast, SD is a renowned high-layer breed, with an average annual egg production exceeding 293–314 eggs (NY/T 3132-2017) [13]. SD ducks were purchased from Baisheng Family Farm (Nanjing, China), and QD ducks were obtained from Mengcheng Qiangying Duck Industry Co., Ltd. (Bozhou, China). Upon arrival, all ducks underwent a two-week acclimatization period under standard management conditions before any procedures began. Ducks were housed in well-ventilated pens with controlled temperature (18–22 °C) and a 14L/10D light cycle. They were provided with unlimited access to duck feed and clean drinking water. Animal health and behavior were monitored daily by trained personnel.

Prior to euthanasia, blood samples (2 mL per duck) were collected from the wing vein using sterile syringes. To minimize stress, ducks were gently restrained by an experienced handler, and the procedure was completed within one minute. After collection, blood was allowed to clot at room temperature for 30 min and then centrifuged at 3000× *g* at 4 °C for 15 min to separate serum. Serum was aliquoted and stored at −20 °C until hormone analysis.

Following blood collection, ducks were euthanized humanely by cervical dislocation performed by specially trained and skilled personnel to ensure instantaneous loss of consciousness and death, in compliance with AVMA (American Veterinary Medical Association) guidelines for poultry. Death was confirmed by the absence of corneal reflex and cessation of breathing.

Immediately after euthanasia, ovarian tissues were rapidly dissected. A representative portion of the ovary was fixed in 4% paraformaldehyde for 24–48 h at 4 °C for subsequent histological processing. The remaining ovarian tissue was snap-frozen in liquid nitrogen within 2–3 min of dissection and then stored at −80 °C until RNA extraction. All tissue handling was performed on ice or at 4 °C to preserve RNA integrity.

### 2.3. Serum Hormone Level Measurement

Duck FSH ELISA Kit (Cat. ANG-E33015D, Angel Gene, Nanjing, China), Duck Estradiol ELISA Kit (Cat. CSB-EQ027953DU, Angel Gene, Nanjing, China), and Duck Progesterone ELISA Kit (Cat. PDEF-123, Angel Gene, Nanjing, China) were used to measure the serum hormone. According to the instructions of each kit, the detection ranges were 0.75–24 mIU/mL for FSH, 40 pg/mL–1000 pg/mL for E2, and 0.2 ng/mL–80 ng/mL for P4. The intra-assay and inter-assay coefficients of variation (CVs) were all <10% and <15%, respectively. All samples from both QD and SD groups were randomized across the same assay plate to minimize inter-plate variation. Absorbance was measured at 450 nm using a microplate reader (BioTek Instruments, Winooski, VT, USA). Hormone concentrations were determined by interpolation from standard curves. All samples were measured in duplicate.

### 2.4. Morphological Observation of Ovary

Paraformaldehyde-fixed ovarian tissues were processed routinely for paraffin embedding. Serial sections of 4–5 μm thickness were cut and stained with Hematoxylin and Eosin (H & E) according to standard protocols. Stained sections were examined under a light microscope to observe follicular development. Follicles were classified into stages according to established morphological criteria: primordial (single layer of squamous granulosa cells), primary (single layer of cuboidal granulosa cells), secondary (two or more layers of granulosa cells, no antrum), and mature (large antral follicle with a clearly defined theca layer). Three non-serial sections were selected from the central region of each ovary. For each section, all follicles in five randomly selected, non-overlapping fields of view (at 5× magnification) were counted and measured. The diameter of each follicle was measured at its widest point using ImageJ (version 1.53t). The mean count and diameter of each view were then calculated for each stage and each individual ovary.

### 2.5. RNA Extraction and Quality Control

Total RNA was extracted from each ovarian sample using TRIzol reagent (Invitrogen, Waltham, MA, USA). RNA concentration and purity were assessed using a Nanodrop-2000 spectrophotometer (Thermo Fisher Scientific, Waltham, MA, USA). RNA integrity was evaluated using an Agilent Bioanalyzer 2100 system (Agilent Technologies, Santa Clara, CA, USA). Only RNA samples with an RNA Integrity Number (RIN) greater than 7.0 were used for subsequent library construction.

### 2.6. PacBio SMRT Long-Read Transcriptome Analysis

#### 2.6.1. PacBio Library Construction and Sequencing

For PacBio sequencing, full-length cDNA was synthesized from high-quality total RNA using the Iso-Seq Express 2.0 kit (Pacific Biosciences, Menlo Park, CA, USA). The SMRTbell library was constructed according to the manufacturer’s instructions. For full-length transcriptome discovery, high-quality cDNA from all six individual samples (three QD and three SD) was pooled in an equimolar ratio to construct a single, pooled SMRTbell library. Library quality and quantity were assessed using Qubit 3.0 (Thermo Fisher Scientific, Waltham, MA, USA) and Agilent Femto Pulse. Qualified pooled library was sequenced on two SMRT Cells of the PacBio Revio sequencer (Pacific Biosciences, Menlo Park, CA, USA) to generate long reads.

#### 2.6.2. PacBio SMRT Data Processing

Raw SMRT sequencing data were processed using the SMRT Link software (v10.0). Circular Consensus Sequences (CCS) were generated, filtered for full-length non-chimeric (FLNC) reads, and clustered to obtain high-quality consensus isoforms. FLNC reads were corrected using LoRDEC (version 0.9) with Illumina short reads and then mapped to the Pekin duck reference genome (GCF_015476345.1) using GMAP (version 2024.07.20) [14].

#### 2.6.3. Identification of Alternative Splicing from PacBio Sequences

AS events were identified and classified using the Astalavista software (version 4.0) [15].

#### 2.6.4. Long Non-Coding RNAs and Open Reading Frames Were Identified from PacBio Sequence

Coding potential of novel transcripts was assessed using CNCI (version 2.0), CPC2 (version 2.0), CPAT (version 3.0.4), and PLEK (version 1.2) tools. The intersection of predictions from all four tools was defined as high-confidence lncRNAs. Open Reading Frames (ORFs) were predicted using TransDecoder (version 5.7.1).

#### 2.6.5. Identification and Analysis of Polyadenylation (APA) Sites

Identification and analysis of polyadenylation (APA) sites were initially identified from the high-quality, full-length SMRT data using PolyA_Skinner (version as per TAPIS suite) and TAPIS tools (version 1.4) [16] to create a comprehensive catalog of potential polyA sites in the duck ovary. To quantify differential APA usage between QD and SD, we then applied DaPars2 (v2.0) to the aligned Illumina short-read data, using the SMRT-derived sites as a reference. Differential APA was called with thresholds set at FDR-adjusted *p*-value < 0.05 and |ΔPDUI| > 0.1.

### 2.7. Illumina RNA-Seq Analysis

#### 2.7.1. Illumina RNA Sequencing Library Construction and Sequencing

For Illumina sequencing, mRNA was enriched from total RNA using oligo(dT) magnetic beads and fragmented. First- and second-strand cDNA were synthesized, followed by end repair, A-tailing, and adapter ligation to construct the sequencing library. Library quality was assessed, and qualified libraries were sequenced on an Illumina NovaSeq 6000 platform (Illumina, San Diego, CA, USA) to generate 150 bp paired-end reads.

#### 2.7.2. Transcriptome Annotation

Novel transcripts and genes were identified by comparing SMRT-derived isoforms with known genome annotations. Functional annotation of novel transcripts was performed by aligning them against the NR, Swiss-Prot, KOG, and KEGG [17,18] databases using Diamond BLASTX (version 2.1.8) [19].

#### 2.7.3. Analysis of Illumina RNA Sequencing Data

Raw Illumina reads were quality-filtered using SOAPnuke (v2.1.0). Clean reads were aligned to the reference genome (GCF_015476345.1) using HISAT2 (v2.2.1) [20], with default parameters except to specify strand-specific information. Gene expression levels were quantified as FPKM (Fragments Per Kilobase of transcript per Million mapped reads). Read counts per gene were generated using featureCounts. Differential expression analysis between QD and SD was performed using DESeq2 (version 1.40.2) [21] on these raw count matrices. Genes satisfying |log2(Fold Change)| > 1 and adjusted *p*-value (padj) < 0.05 were considered differentially expressed (DEGs). FPKM values were calculated separately for visualization and comparative assessment of expression levels.

#### 2.7.4. Functional Enrichment Analysis

Gene Ontology (GO) and KEGG pathway enrichment analyses were performed for DEGs and genes with significant APA events using the clusterProfiler package (v4.6.2) [22] in R (version 4.3.1). Terms with an FDR-adjusted *p*-value (q-value) < 0.05 were considered significantly enriched.

#### 2.7.5. qPCR Validation

qPCR was performed using SuperStar Universal SYBR Master Mix (Cat. CW33360M, CWBIO, Nanjing, China) on a Pangu real-time PCR system. Primer specificity was confirmed by a single peak in melt-curve analysis, and amplification efficiency (90–110%) was validated using standard curves. The *ACTB* (β-actin) gene was used as an internal control as its expression was stable across all samples in our RNA-seq data (FPKM coefficient of variation < 10%). The seven genes (*H3*, *CENPA*, *ECM29*, *UPK1B*, *HMGCS1*, *SOD1*, *SCD*) were selected to validate the RNA-seq platform across a range of expression levels and gene functions. Relative expression levels were calculated using the 2^−ΔΔCt^ method.

### 2.8. Integrated Analysis of DEGs and APA Events

Overlapping genes between DEGs and genes with significant APA events (shortened or lengthened 3′UTR) were identified to screen for key candidates potentially regulated at both transcriptional and post-transcriptional levels.

### 2.9. Statistical Analysis

For serum hormone data and follicular measurements, statistical significance between QD and SD groups was determined using an independent-samples *t*-test in GraphPad Prism (Version 9.0). Prior to *t*-tests, data distribution normality was tested using the Shapiro–Wilk test. F-test was used to assess the data variances. Data are presented as mean ± standard deviation (SD), and differences were considered statistically significant at *p* < 0.05.

## 3. Results

### 3.1. Comprehensive Assessment of Serum Hormones and Ovarian Structures

The serum concentrations of key reproductive hormones (follicle-stimulating hormone (FSH), estradiol (E_2_), and progesterone (P_4_)) were quantified.

**Figure 2:**
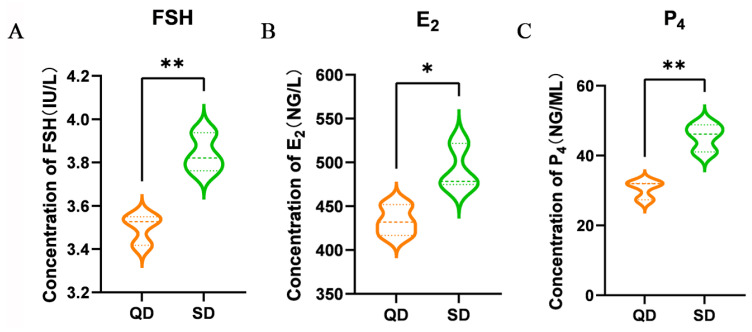
The serum hormone levels in Qiangying Duck (QD, meat-type) and Shaoxing Duck (SD, laying-type). (**A**) Follicle-stimulating hormone (FSH) levels measured by ELISA (n = 6). (**B**) Estradiol (E2) levels measured by ELISA (n = 6). (**C**) Progesterone (P4) levels measured by ELISA (n = 6). Horizontal dashed lines indicate the mean value for each breed. Significant differences are denoted as follows: * (*p* < 0.05), ** (*p* < 0.01).

SD exhibited significantly higher levels of FSH (*p* < 0.01), E_2_ (*p* < 0.01), and P_4_ (*p* < 0.01) compared to QD (Figure 2A–C).

**Figure 3:**
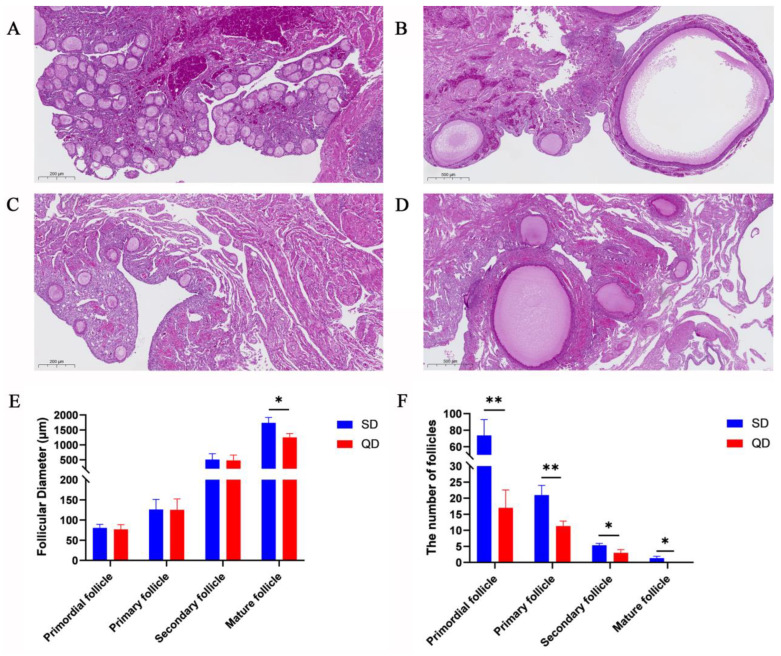
Histomorphological characteristics of duck ovarian tissues. (**A**) Primordial follicles in SD ovaries. (**B**) Primary follicles, secondary follicles, and mature follicles in SD ovaries. (**C**) Primordial follicles in QD ovaries. (**D**) Primary follicles, secondary follicles in QD ovaries. (**E**) The diameter of follicles at different developmental stages (primordial, primary, secondary, and mature follicles). (**F**) The number of follicles at different developmental stages (primordial, primary, secondary, and mature follicles). * indicates a significant difference between SD and QD (*p* < 0.05). ** indicates a significant difference between SD and QD (*p* < 0.01). Data are presented as mean ± standard deviation (SD) from n = 3 independent biological replicates (ducks) per breed. QD: Qiangying Duck, meat-type; SD: Shaoxing Duck, laying-type.

In addition, H & E staining was performed on the ovarian tissue to compare the quantity and size of the follicles (Figure 3), which presents a direct visualization of follicle development between QD and SD. It was found that primordial follicles, secondary follicles, and mature follicles are more abundant in the SD ovary than in the QD ovaries. The diameter and number of follicles at different developmental stages were measured and statistically analyzed. The results demonstrated that only the average diameter of mature follicles per field of view in the SD group was significantly larger than that in the QD group (*p* < 0.05), while no significant differences were observed in follicles at other stages. However, the average number of follicles per field of view at all stages in the SD group was significantly greater than that in the QD group.

### 3.2. SMRT-Sequencing Identified Novel Transcriptional Dynamics in Duck Ovary

To comprehensively characterize the transcriptomes of QD and SD duck ovaries, we employed PacBio Single-Molecule Real-Time (SMRT) sequencing. For each breed, full-length cDNA from the three biological replicates was pooled to generate a breed-specific library for sequencing. The raw data were processed using the SMRT Link software and the Iso-Seq analysis pipeline to obtain high-quality full-length transcript sequences.

Initial data processing yielded a total of 7,548,345 and 7,888,301 Circular Consensus Sequences (CCS) for QD and SD, respectively. The average lengths of these CCS reads were 1731 bp for QD and 1878 bp for SD, with N50 lengths of 1826 bp and 1954 bp, respectively (Table S1, Figure S1), indicating the generation of long and high-quality sequencing data. Subsequent classification identified 7,547,897 and 7,887,788 full-length (FL) reads, 7,539,068 and 7,875,476 full-length non-concatemer (FLNC) reads, and 7,534,547 and 7,870,100 FLNC reads with polyA tails for QD and SD, respectively (Table S2). The high proportion of FLNC reads demonstrates the exceptional capability of SMRT sequencing in capturing complete transcript isoforms.

**Figure 4:**
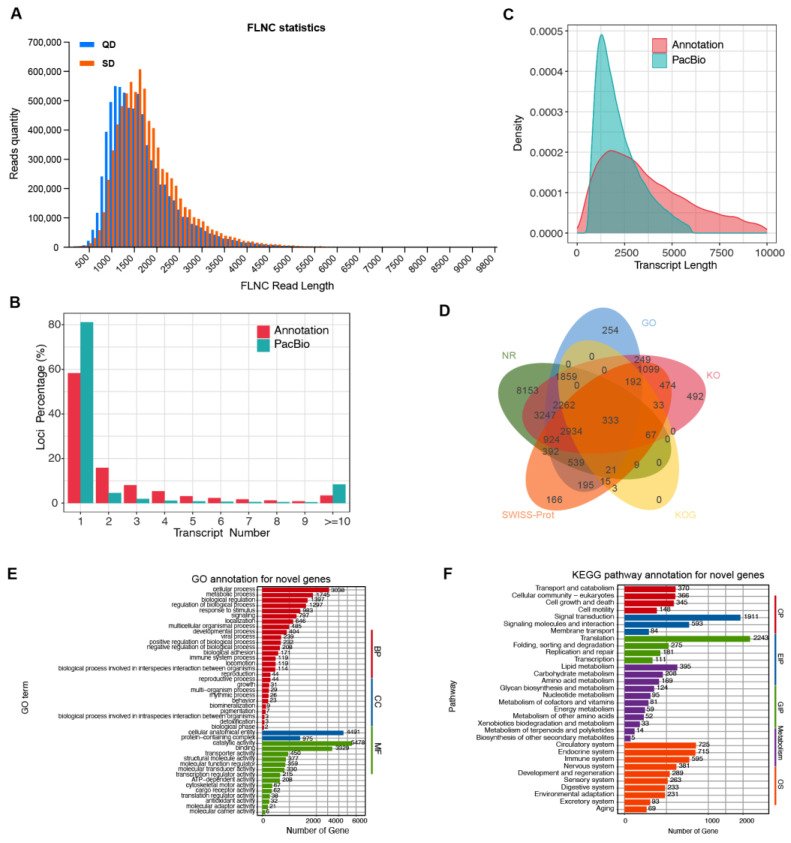
SMRT-sequencing reveals novel transcriptional dynamics in duck ovaries. (**A**) FLNC distribution statistics based on their read length. (**B**) The loci distribution comparison between known annotation and this Pacific Biosciences (PacBio) SMRT-sequencing, based on the transcript number. (**C**) The density distribution of the transcript length in known annotation and this SMRT-sequencing. (**D**) The annotation intersection of novel genes for 5 databases, including NR, GO, KO, KOG, and SWISS-Prot. (**E**) GO annotation for novel genes. (**F**) KEGG pathway annotation for novel genes. Bar length represents the enrichment significance (−log10 of the raw *p*-value). Significantly enriched terms were selected based on a False Discovery Rate (FDR) < 0.05. For clarity, the top 42 most significantly enriched terms (based on *p*-value) within each GO category (Biological Process, BP; Cellular Component, CC; Molecular Function, MF) are displayed. Abbreviations in the figure: OS, ovary-specific; EIP, estrogen-induced process; CP, cellular process; GIP, gene interaction pathway.

The FLNC reads were then clustered to remove redundancy. To ensure high-confidence isoforms, consensus isoforms were required to be supported by a minimum of 3 full-length reads and were filtered to remove potential artifacts from internal priming or degraded mRNA. As summarized in Table S3, this step resulted in 7,534,547 FLNC reads for QD (average length: 1622 bp; N50: 1737 bp) and 7,870,100 for SD (average length: 1767 bp; N50: 1864 bp), with length ranges from 50 bp to approximately 9.7 kb for both varieties. The clustering effectively consolidated the data while preserving the full-length diversity of the transcriptomes. Interestingly, the quantity of longer FLNC read length (greater than 1500 bp) in SD is higher than that in QD (Figure 4A).

Considering the high base error rate of single-molecule sequencing technology, LoRDEC was used to further correct the FLNC data. Correction was evaluated by PID (Percentage of Identification); that is, the FLNC sequences before and after correction were aligned into reference genome (Pekin duck reference genome with GCF_015476345.1) by GMAP. The mean entire PID of post-correct FLNC was 97.60% (Table S4), and the high quality mapped FLNC percentage was 90.41% (Table S5). Based on merge FLNC mapping position, gene loci and isoform of whole genome could be identified. Here, a total of 521,034 isoforms and 118,801 loci were respectively identified (Table 1). Among them, loci with only one transcript were dominant, more than 80% (Figure 4B). Moreover, the length of transcripts was mainly around 1000 bp, and the density of length dominant transcripts was much higher than that of previously annotated transcripts (Figure 4C). Overall, our long-read sequencing data have improved duck genome annotation and ovary transcriptional landscape.

Alignment of genes and isoforms generated from SMRT-seq with known annotation of the reference genome enabled the identification of novel genes and isoforms. Here, a comprehensive annotation against the reference genome revealed a substantial expansion of the transcriptional landscape (Table S5). We identified a total of 118,801 gene loci and 521,034 transcript isoforms. As de-tailed in Table S5, the majority of novel discoveries were novel isoforms of previously annotated genes (392,026 isoforms from 20,354 known loci). Furthermore, we discovered 111,278 novel transcript isoforms originating from 97,571 novel gene loci not present in the current annotation. For downstream functional analysis, we focused on this set of 97,571 novel loci (and their 111,278 isoforms). All newly obtained transcript sequences were aligned against five databases: NR, GO, KO, KOG/COG, and Swiss-Prot. Moreover, these novel genes and isoforms could be primarily annotated by NR, GO, and KEGG pathway databases (Figure 4D, Table 2, and Table S6). For GO annotation, these novel genes were primarily enriched in catalytic activity, cellular anatomical entity, and cellular process (Figure 4E). For KEGG pathway annotation, these novel genes were primarily enriched in Translation and Signal transduction (Figure 4F).

### 3.3. Identification of lncRNA and ORF Prediction for Novel Isoforms

**Figure 5:**
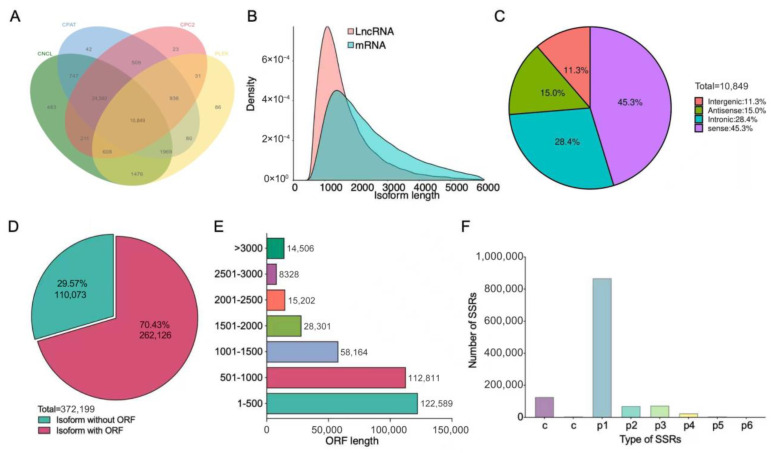
The identification and characterization of long non-coding RNAs (lncRNAs) and open reading frames (ORFs) for novel isoforms in duck ovaries. (**A**) The number of high-confidence lncRNAs identified. (**B**) The length distribution of lncRNAs and mRNAs. (**C**) The classification of lncRNAs based on genomic context. (**D**) The prediction of complete and incomplete ORFs from novel isoforms. (**E**) The length distribution of predicted ORFs. (**F**) The frequency distribution of simple sequence repeat (SSR) types identified by MISA. Abbreviations: CNCI, Coding-Non-Coding Index; CPC2, Coding Potential Calculator 2; CPAT, Coding Potential Assessment Tool; PLEK, Predictor of Long non-coding RNAs and messenger RNAs based on an improved k-mer scheme.

To characterize the coding potential of novel isoforms, we systematically identified long non-coding RNAs (lncRNAs). Putative lncRNAs were filtered using the following stringent criteria: (1) transcript length ≥ 200 bp; (2) containing two or more exons; (3) lack of coding potential as predicted by all four tools (CNCI, CPC2, CPAT, and PLEK); (4) not overlapping with any known protein-coding exon in the sense orientation. We further removed transcripts with first or last exon length < 15 bp and removed transcripts with an average expression level (FPKM) across all six samples below 0.1. Application of these filters yielded 10,849 high-confidence lncRNAs (Figure 5A). The length of the identified lncRNAs was mainly about 1100 bp, while the length of the mRNA was mainly about 1400 bp (Figure 5B). More than 45% lncRNAs could be classified as sense lncRNAs (Figure 5C). Subsequently, the predicted lncRNAs were filtered out from the novel isoforms. Moreover, the ORFs predicted by transDecoder were used to further characterize the possible action forms of the novel isoforms identified in duck ovary. Here, 372,199 isoforms were used for ORF prediction, of which more than 70% were predicted to possess complete ORFs (Figure 5D), indicating a high coding potential of these novel isoforms within the ovarian transcriptome. The length of these predicted ORFs was mainly within 1000 bp (Figure 5E). Additionally, SSR analysis performed with MISA software revealed a predominance of mono-nucleotide repeats (Figure 5F).

### 3.4. Post-Transcriptional Alternative Structural Regulation of Novel Isoforms

**Figure 6:**
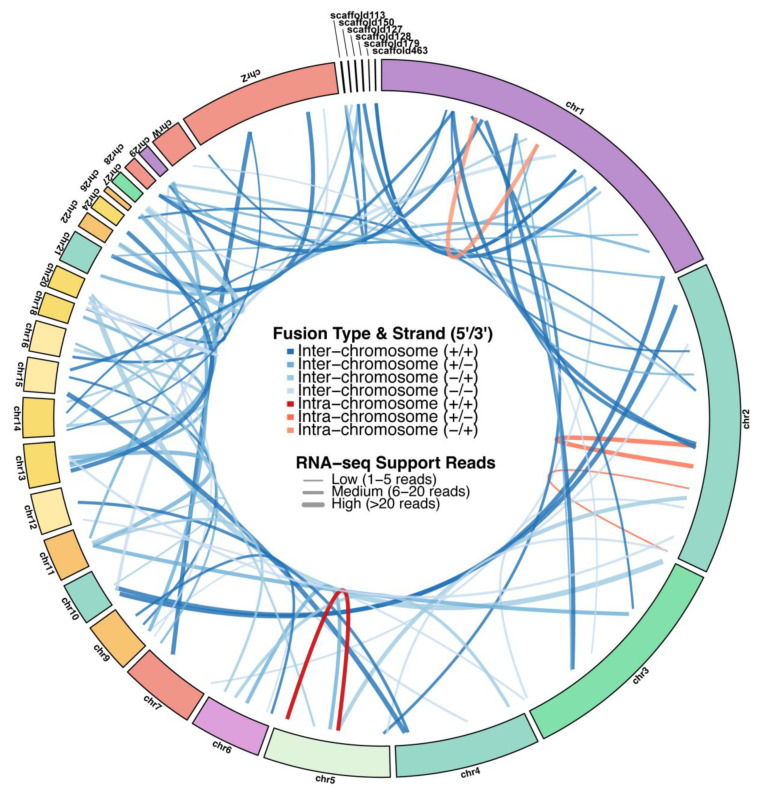
Genomic distribution of fusion genes identified in duck ovary. The pie chart illustrates the proportion of intra-chromosomal (blue, n = 4) and inter-chromosomal (orange, n = 76) fusion genes, as validated by SMRT-seq and RNA-seq data.

Alternative splicing (AS) analysis was performed to profile post-transcriptional regulatory complexity in duck ovaries. A total of 287,068 AS events were identified across all samples, which could be classified into 6 types, including exon skipping (ES), alternative donor site (AD), alternative acceptor site (AA), intron retention (IR), mutually exclusive exons (MEE), and other complex events (Figure S2). Among these, ES was the most predominant type, which is consistent with its recognized status as the most common form of AS in eukaryotic mRNAs and validates the reliability of our splicing annotation. The prevalence of ES highlights its potential significant role in generating transcriptome diversity relevant to ovarian function in ducks. In addition, 88 high-confidence fusion transcript events were identified from the SMRT-seq data, each supported by both long reads and independent Illumina short reads spanning the fusion junction (Table S7). These were detected under stringent criteria and are presented as a catalog of potential post-transcriptional events for future investigation. The 88 fusion gene events could be annotated as 80 fusion genes, including 4 intra-chromosome fusion genes and 76 inter-chromosome fusion genes (Figure 6).

### 3.5. Analysis of Alternative Polyadenylation Sites

**Figure 7:**
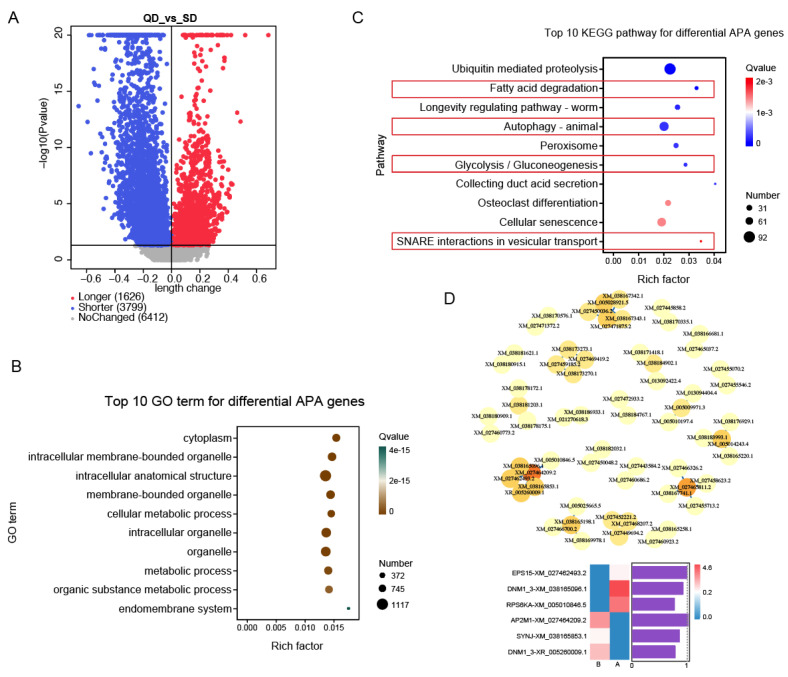
An analysis of alternative polyadenylation (APA) in duck ovary. (**A**) The global profiling and statistical significance of identified APA sites. The dashed line indicates the threshold (*p*-value < 0.05) for significant 3′ UTR length change. (**B**) Gene Ontology (GO) enrichment analysis of genes with significant APA events. (**C**) The KEGG pathway enrichment analysis of genes exhibiting significant APA. Key enriched pathways are highlighted. (**D**) The transcript interaction network constructed from APA analysis, depicting 49 potential interactions among 62 transcripts. The graph below the network is the heatmap of the expression and the purple columns present normalization value of 3’UTR length difference; the value greater than 1 means longer while value. less than 1 means shorter. For clarity, the top 10 significantly enriched terms (based on *p*-value) within each GO category (Biological Process, BP; Cellular Component, CC; Molecular Function, MF) are displayed. QD: Qiangying Duck, meat-type; SD: Shaoxing Duck, laying-type.

Alternative polyadenylation (APA) analysis was conducted based on the SMRT sequencing data to investigate post-transcriptional regulation at the 3′ end of transcripts. A global profile of APA sites was generated, and this identified a total of 11,837 genes containing APA on their own 3′ UTR. *p*-value < 0.05 was set as the threshold for 3′ UTR length change based on APA sites (Figure 7A).

Compared to QD, SD exhibited significant 3′ UTR shortening in 3799 genes (FDR < 0.05, Δ PDUI < 0.1) and lengthening in 1626 genes, indicating widespread APA-mediated remodeling of transcript 3′ ends. To elucidate the biological implications of the identified APA differences between QD and SD, GO and KEGG pathway enrichment analyses were performed on genes exhibiting significant APA events, respectively. GO and KEGG enrichment analyses revealed that genes with differential APA were significantly enriched in terms related to organelle development and metabolic processes (Figure 7B), and pathways including SNARE interactions in vesicular transport, glycolysis/gluconeogenesis, and fatty acid degradation (Figure 7C). In addition, to explore the functional coordination among genes with significant APA, we constructed a protein–protein interaction network using high-confidence interaction data from the STRING database (combined score > 0.7) for the corresponding proteins. This analysis revealed 49 high-confidence interactions among 62 transcripts (nodes) (Figure 7D). Notably, topological analysis identified several highly connected hub nodes within this network. Among them were genes encoding key components of the SNARE complex (e.g., VAMP8, SNAP23), which are essential for intracellular vesicle trafficking. More importantly, intracellular vesicle trafficking is a process fundamental to nutrient transport, steroid precursor delivery, and cell signaling during follicular development. The centrality of such regulators in the APA-associated network suggests that post-transcriptional 3′ UTR remodeling may converge on core functional modules to coordinately influence ovarian physiology.

### 3.6. Identification and Functional Profiling of Differentially Expressed Genes

**Figure 8:**
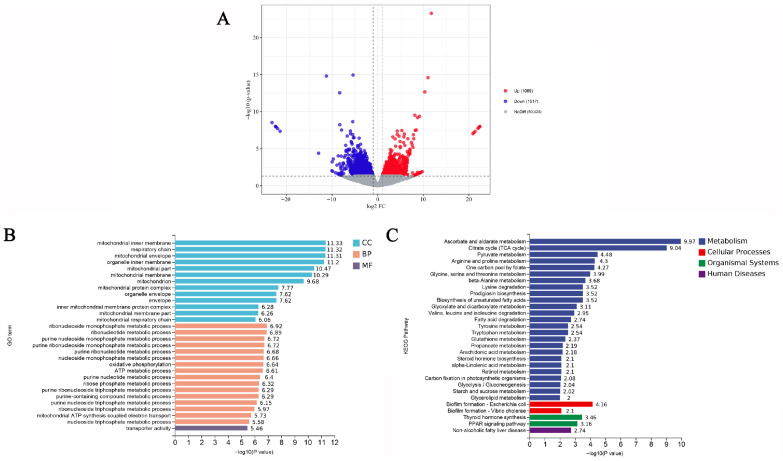
The identification and functional analysis of differentially expressed gene. (**A**) A volcano plot of differentially expressed genes between QD and SD groups. Vertical dotted lines typically indicate the fold change threshold (|log_2_FC| > 1), while a horizontal line represents the statistical significance threshold (*p*-value < 0.05). (**B**) GO annotation for the differentially expressed gene. (**C**) KEGG pathway annotation for the differentially expressed gene. The bar length represents the enrichment significance (−log10 of the raw *p*-value). Significantly enriched terms were selected based on a False Discovery Rate (FDR) < 0.05. For clarity, the top 30 significantly enriched terms (based on *p*-value) within each GO category (Biological Process, BP; Cellular Component, CC; Molecular Function, MF) are displayed. QD: Qiangying Duck, meat-type; SD: Shaoxing Duck, laying-type.

A total of 2606 differentially expressed genes (DEGs) were identified between SD and QD, including 1089 up-regulated genes and 1517 down-regulated genes (Figure 8A). To gain insight into the biological functions of the identified DEGs between SD and QD, we performed systematic GO and KEGG pathway enrichment analyses. The GO enrichment analysis revealed that these genes were significantly enriched in terms associated with mitochondrial structure and function, including mitochondrial inner membrane, respiratory chain, organelle envelope, and oxidative phosphorylation (Figure 8B). Additionally, multiple metabolic processes such as ribonucleotide metabolic process, purine-containing compound metabolic process, and ATP metabolic process were also prominently enriched, suggesting a central role of energy metabolism and nucleotide biosynthesis in the observed transcriptional changes. The KEGG pathway analysis further indicated that these genes were involved in key metabolic pathways, including the citrate cycle (TCA cycle), pyruvate metabolism, glycolysis/gluconeogenesis, and fatty acid degradation (Figure 8C). Other enriched pathways such as arginine and proline metabolism, glycine, serine and threonine metabolism, and glutathione metabolism also highlighted the active involvement of amino acid and antioxidant processes. These findings indicate that the genes with differential expression may be involved in the regulation of energy supply, nutrient metabolism, and redox balance during follicle development and maturation. These processes are crucial for the cell development and functional maintenance of both the QD group and the SD group. A list of the top 20 significantly upregulated and downregulated DEGs is provided in Supplementary Table S8.

### 3.7. Quantitative Real-Time Polymerase Chain Reaction

**Figure 9:**
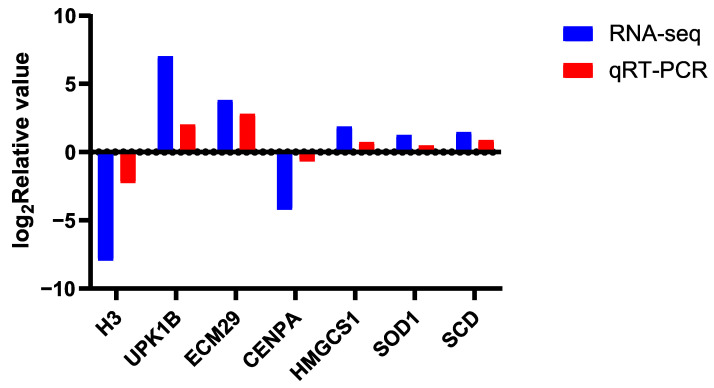
RNA-Seq validation using qRT-PCR.

To validate the gene expression patterns identified by the RNA-Seq analysis, we selected seven genes for quantitative real-time PCR (qRT-PCR) analysis. The candidate genes included Histone H3 (*H3*), Centromere Protein A (*CENPA*), Extracellular Matrix Protein 29/Proteasome Scaffold Protein (*ECM29*), Uroplakin 1B (*UPK1B*), 3-Hydroxy-3-Methylglutaryl-CoA Synthase 1 (*HMGCS1*), Superoxide Dismutase 1 (*SOD1*), and Stearoyl-CoA Desaturase (*SCD*), Specific information is presented in Table S9. The expression patterns detected for all selected genes were highly consistent with the RNA-Seq results (Figure 9), thereby confirming the reliability of our transcriptomic data.

### 3.8. Conjoint Analysis

To comprehensively elucidate the complexity of gene expression regulation, we performed an intersection analysis between DEGs and genes exhibiting significant APA events categorized into shortening and lengthening groups. By correlating gene expression levels with APA site usage patterns, we investigated whether expression differences between samples were associated with APA events, thereby exploring potential post-transcriptional regulatory mechanisms.

**Figure 10:**
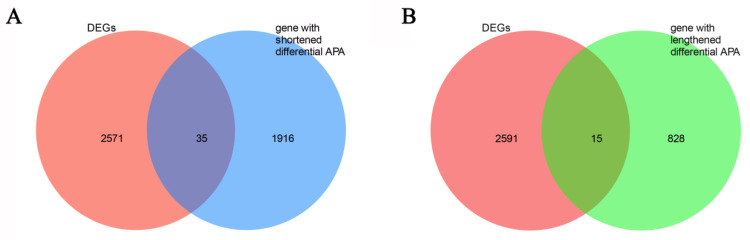
Venn diagram showing the overlap between differentially expressed genes (DEGs) and genes with significant alternative polyadenylation (APA) events. (**A**) Intersection between DEGs and genes exhibiting shortened 3′UTR APA. A total of 35 genes are shared between the two sets. (**B**) Intersection between DEGs and genes exhibiting lengthened 3′UTR APA. A total of 15 genes are shared between the two sets.

We identified 35 overlapping genes between DEGs and those with shortened 3′ UTRs (Figure 10A, Table S10). Among these 35 overlapping genes, we prioritized those with established or strongly implicated roles in ovarian development, steroidogenesis, or lipid metabolism based on extensive reviews. Among them, *HMGCS1* and *DHCR24* are directly involved in cholesterol biosynthesis, which may influence steroid hormone production. Additionally, their antioxidant functions could also help protect ovarian cells. *SCD*, a central enzyme in lipid metabolism, is critical for ovarian steroidogenesis and thus ovarian development. HRAS acts as an upstream regulator of key signaling pathways such as MAPK/ERK, broadly participating in cell proliferation, differentiation, and survival, with potential regulatory roles at multiple stages of follicular development. *NR5A1* serves as a master regulator of steroidogenic tissue development and function. All of these genes may collectively influence ovarian development through related biological processes.

In addition, 15 genes were identified between DEGs and those with 3′UTR lengthening (Figure 10B, Table S10). Similarly, from the 15 genes overlapping with 3′UTR lengthening, we focused on genes with key functions in antioxidant defense and hormone signaling. *SOD1*, a key antioxidant enzyme, catalyzes the conversion of superoxide radicals into hydrogen peroxide, thereby protecting ovarian cells from oxidative stress. *PGR*, the progesterone receptor, mediates the effects of progesterone on ovulation-related gene expression, oocyte maturation, and corpus luteum function. Therefore, these overlapping genes represent candidate modulators of ovarian development, likely acting via their distinct molecular mechanisms.

## 4. Discussion

This study provides a comprehensive, integrated long-read transcriptome atlas of the duck ovary, substantially expanding genomic annotation and revealing extensive post-transcriptional complexity through widespread AS and APA. The comparative analysis between meat-type (QD) and egg-type (SD) ducks uncovered distinct APA and gene expression profiles, with functional enrichment underscoring critical roles for energy metabolism, steroidogenesis, and antioxidant defense. These findings establish a foundational molecular basis for their divergent reproductive performance and highlight the importance of post-transcriptional regulation in shaping ovarian function.

Alternative splicing (AS) and alternative polyadenylation (APA) represent two crucial mechanisms of post-transcriptional regulation in gene expression [11]. Alternative splicing (AS) significantly enhances transcriptomic and proteomic diversity by enabling a single gene to generate multiple mRNA isoforms through differential splicing of exons and introns [23,24]. Our results demonstrate that exon skipping (ES) represents the most prevalent AS event in duck ovarian tissues, which aligns with findings in most higher eukaryotes. ES events typically exhibit tissue-specific and developmental stage-specific patterns, allowing for the precise regulation of protein functions required under specific physiological conditions. This regulatory mechanism serves as a critical genetic basis for important economic traits such as growth, reproduction, and stress resistance [25,26]. Furthermore, the distinct signatures of ES events in sequencing data facilitate their detection and quantification, making ES an ideal focus for investigating transcriptome complexity using high-throughput technologies [27]. Consequently, ES is recognized as a fundamental mechanism for expanding genomic coding capacity, enhancing proteomic diversity, and increasing regulatory complexity [28].

APA represents another widespread post-transcriptional regulatory mechanism, where the selection of different polyadenylation sites enables a single gene to produce transcripts with varying 3′ UTRs [29]. The 3′ UTR plays crucial roles in mRNA stability, translation efficiency, and subcellular localization [11,30].

Our results resonate with and extend findings from other poultry species. Similar to transcriptomic studies in high-laying chickens, pathways central to energy metabolism and steroidogenesis were markedly enriched in the laying-type duck [31], reaffirming their conserved role in supporting high egg output. While APA events occurring in chicken have been noted to be growth [32], the novel contribution of our work lies in revealing the exceptional scale and phenotypic association of APA in the duck ovary. Their widespread occurrence and strong linkage to divergent laying performance, as documented here, suggest that post-transcriptional 3′ UTR remodeling may be a potential regulatory strategy in ducks.

The functional significance of these widespread APA events is underscored by their specific targeting of genes central to ovarian physiology. We find each key gene with shortened 3′ UTRs tends to be upregulated [e.g., *HMGCS1* (log2FC: 1.87, ΔPDUI: −0.167), *SCD* (log2FC: 1.47, ΔPDUI: −0.11)], and vice versa. Our integrated analysis revealed several major candidate genes that form a cohesive molecular network enhancing ovarian function in SD ducks. At the core of this network are genes governing steroidogenic precursor supply. *HMGCS1*, encoding a rate-limiting enzyme in cholesterol synthesis, was upregulated and exhibited 3′ UTR shortening in SD ducks. Given that cholesterol is the essential precursor for all steroid hormones [33,34], this coordinated change provides a direct molecular link to the elevated serum estradiol and progesterone levels we observed in SD ducks (Figure 1). Consistent with this reinforced biosynthetic pathway, *DHCR24* is the final enzyme in cholesterol biosynthesis with additional antioxidant roles [35]. This dual-function enzyme may thus concurrently support high steroid output and mitigate associated oxidative stress in the metabolically active SD ovary. Beyond steroidogenesis, efficient lipid metabolism and precise cellular signaling are crucial for follicular development [36]. *SCD*, a key lipogenic enzyme, was also upregulated with 3′ UTR shortening in SD ducks. Its role in generating monounsaturated fatty acids is vital for membrane integrity and signaling lipid production, processes essential for follicular growth and oocyte maturation [37]. Signaling hubs such as *HRAS* also exhibited differential regulation, potentially serving to coordinate growth with metabolic demands. As a central node in the MAPK/ERK and PI3K/AKT pathways, differential *HRAS* expression could adjust the responsiveness of ovarian somatic cells to hormonal stimuli [38], thereby modulating the distinct follicular development dynamics observed between the breeds (Figure 2). This rewiring of metabolic and signaling pathways is likely orchestrated by master transcriptional regulators. *NR5A1* (Steroidogenic Factor 1) is a master transcriptional regulator of genes critical for gonadal development and steroid hormone synthesis and was identified as a candidate [39,40]. Its differential regulation suggests a fundamental re-programming of the ovarian transcriptome in SD ducks. *SOD1*, a crucial antioxidant enzyme, was differentially regulated. By protecting ovarian cells from oxidative damage associated with frequent ovulation [41], *SOD1* may safeguard oocyte quality and follicular health in the high-layer breed. Finally, for the precise execution of reproductive cycles, hormone sensitivity is essential. We found evidence for post-transcriptional tuning of *PGR*, the progesterone receptor. As progesterone is critical for ovulation and corpus luteum function, breed-specific APA of *PGR* could influence the efficiency of ovulation-related gene expression [42,43], thus affecting a final crucial step linking ovarian molecular events to the tangible output of egg laying. The distinct expression and APA profiles of these genes highlight their collective importance in regulating the divergent ovarian physiology and reproductive performance between meat-type and egg-type ducks.

In summary, this study systematically elucidates the crucial roles of AS and APA in the transcriptional regulation of duck ovaries, identifying key genes—including *HMGCS1*, *DHCR24*, *SCD*, *HRAS*, *NR5A1*, *SOD1*, and *PGR*—that influence ovarian development and function. These findings provide novel insights into the post-transcriptional regulatory network underlying avian reproductive traits and establish a theoretical foundation for subsequent functional validation and breeding applications.

While this study provides a foundational atlas and novel insights, several limitations should be acknowledged. First, the modest sample size (n = 3 per group), though sufficient for identifying large-effect transcriptional changes, may limit the detection of subtle or highly variable regulatory events and warrants validation in larger cohorts. Second, our work is primarily correlative and descriptive; the functional necessity of the identified APA events and candidate genes (e.g., *SCD*, *HMGCS1*) for the laying phenotype remains to be experimentally validated. Third, this study was designed to comprehensively characterize APA. Consequently, the analysis of alternative splicing (AS), while confirming widespread transcriptome complexity, remains descriptive and was not powered for breed-specific comparisons or functional synergy with APA. Future research should prioritize the following: (1) functional dissection of key APA sites using in vitro reporter assays or in vivo genome editing in model systems to confirm their impact on gene expression and ovarian physiology and (2) the systematic investigation of breed-specific AS regulation and its potential interplay with APA, using larger cohorts and dedicated analytical tools.

## 5. Conclusions

This study integrated PacBio SMRT and Illumina sequencing to construct a high-resolution transcriptomic atlas of the duck ovary, substantially expanding genomic annotations. We revealed extensive post-transcriptional regulatory complexity and identified significant breed-specific differences in alternative polyadenylation (APA) patterns and gene expression between meat-type (QD) and egg-type (SD) ducks. Functional enrichment analyses highlighted the specific enrichment of key pathways, including energy metabolism, steroid synthesis, and antioxidant defense in SD ducks. Through integrated analysis, we identified a set of major candidate genes (including *HMGCS1*, *DHCR24*, *SCD*, *HRAS*, *NR5A1*, *SOD1*, and *PGR)* could contribute to the divergent ovarian physiology between the breeds. These findings provide fundamental insights into post-transcriptional regulation in avian reproduction and establish a valuable resource and candidate gene list for future functional validation and genetic improvement of laying traits.

## Figures and Tables

**Table 1:** Identification statistics of loci and isoform.

Feature	Annotation.loci.len	PacBio.loci.len
Loci	24,104	118,801
Loci < 1K	3436 (14.25%)	16,046(13.51%)
Loci 1–2K	5587 (23.18%)	48,868(41.13%)
Loci 2–3K	4621 (19.17%)	24,432(20.57%)
Loci > 3K	10,460 (43.40%)	29,455(24.79%)
Total isoform	59,533	521,034

**Table 2:** Information of function annotation.

Database	Novel Genes	Novel Isoforms
Total	97,571 (100.00%)	111,278 (100.00%)
NR	25,155 (25.78%)	29,482 (26.49%)
GO	9952 (10.20%)	11,513 (10.35%)
KO	12,306 (12.61%)	14,332 (12.88%)
KOG	673 (0.69%)	949 (0.85%)
Swiss-Prot	7396 (7.58%)	9158 (8.23%)
Unannotated	72,233 (74.03%)	81,584 (73.32%)

## Data Availability

The raw data supporting the conclusions of this article will be made available by the authors on request.

## Supplementary Materials

The following supporting information can be downloaded at https://www.mdpi.com/article/10.3390/ani16020313/s1. Figure S1. The predictive analysis of novel transcript. (A) The distribution of CCS lengths of QD. (B) The distribution of CCS lengths of SD. QD: Qiangying Duck, meat-type; SD: Shaoxing Duck, laying-type; Figure S2. Analysis of AS events. (A) Classification diagram of AS events. (B) A bar chart with a variable number of AS event types. (C) Pie chart of the proportion of each type of AS event; Table S1. number of CCS identified; Table S2. Type and number of CCS identified; Table S3. Type and number of FLNC identified; Table S4. GMAP mapping statistics; Table S5. Statistics of novel genes and novel isoforms; Table S6. Summary of functional annotation for predicted genes; Table S7. Summary of fusion transcripts identified by FLNC long-read sequencing; Table S8. A list of the top 20 significantly upregulated and downregulated DEGs; Table S9. Primer sequences for qPCR. Table S10. Gene list overlapping with 3’UTR
