# Supplementary material for: A Comparative Analysis of Transcriptome-Wide Differential Gene Expression and Alternative Polyadenylation in the Ovaries of Meat Ducks and Laying Ducks
Source: Animals (Basel). 2026 Jan 20;16(2):313. doi: 10.3390/ani16020313 (PMC12837750; doi:10.3390/ani16020313)
Supplement: Supplementary file 1 [file animals-16-00313-s001.zip › animals-4068500-supplementary.pdf]

Supplementery information

Table S1. number of CCS identified.

Sample	Total_bases	Total_number	Minimum_length	Average_length	Maximum_length	N50
QD	13063528079	7548345	126	1731	14028	1826
SD	14807231196	7888301	124	1878	11872	1954

QD: Qiangying Duck, meat-type; SD: Shaoxing Duck, laying-type.

Table S2. Type and number of CCS identified.

Sample	num_reads_fl	num_reads_flnc	num_reads_flnc_polya
QD	7547897	7539068	7534547
SD	7887788	7875476	7870100

QD: Qiangying Duck, meat-type; SD: Shaoxing Duck, laying-type.

Table S3. Type and number of FLNC identified.

Sample	Total_bases	Total_number	Minimum_length	Average_length	Maximum_length	N50
QD	12219931115	7534547	50	1622	9775	1737
SD	13906383271	7870100	50	1767	9597	1864

QD: Qiangying Duck, meat-type; SD: Shaoxing Duck, laying-type.

Table S4. GMAP mapping statistics.

Feature	Precorr	Postcorr	Merge
unmap	30,028(1.76%)	29,810(1.75%)	29,705(1.74%)
multiple-best	3,932(0.23%)	3,982(0.23%)	3,290(0.19%)
low pid	147,615(8.66%)	175,995(10.32%)	130,515(7.66%)
high quality map	1,523,147(89.35%)	1,494,935(87.69%)	1,541,212(90.41%)

Table S5. Statistics of novel genes and novel isoforms.

Categories	# of loci	# of isoform
annotated gene annotated + novel isoform	21,230	409,756
annotated gene novel isoform	20,354	392,026
novel gene novel isoform	97,571	111,278

Total	118,801	521,034

Table S6. Summary of functional annotation for predicted genes.

Gene_ID	GO_ID	GO_term	KEGG_Pathway	Pathway_ID	KOG_annotation	Swiss-Prot_ID	Swiss-Prot_annotation
NC_009684.1.1	-	-	-	-	-	-	-
NC_009684.1.2	GO:0016021; GO:0008137; GO:0005743	integral component of membrane;NADH dehydrogenase (ubiquinone) activity;mitochondrial inner membrane	Oxidative phosphorylation; Thermogenesis;Retrograde endocannabinoid signaling	ko00190,ko04714,ko04723	NADH dehydrogenase subunit 1	sp|O21397|NU1M_STRCA	NADH-ubiquinone oxidoreductase chain 1 OS=Struthio camelus OX=8801 GN=MT-ND1 PE=3 SV=2
NC_009684.1.3	GO:0016021; GO:0042773; GO:0008137; GO:0070469; GO:0005743	integral component of membrane;ATP synthesis coupled electron transport; NADH dehydrogenase (ubiquinone) activity;respirasome; mitochondrial inner membrane	Oxidative phosphorylation; Thermogenesis;Retrograde endocannabinoid signaling	ko00190,ko04714,ko04723	-	sp|Q35813|NU5M_STRCA	NADH-ubiquinone oxidoreductase chain 5 OS=Struthio camelus OX=8801 GN=MT-ND5 PE=3 SV=2
NC_051772.1.10	-	-	-	-	-	-	-

NC_0517 72.1.1000	-	-	-	-	-	-	-

Supplementary Table Legend: This table presents only 5 pieces of information, including the following information: Gene ID, Gene Ontology (GO) terms and IDs, KEGG Pathway IDs and associated pathways, KOG annotations, and Swiss-Prot IDs with protein descriptions. The symbol "--" indicates no significant match or annotation was available.

Table S7. Summary of fusion transcripts identified by FLNC long-read sequencing.

FLNC_id	5'_gene	5'_chr_id	5'_chr_start_end	3'_gene	3'_chr_id	3'_chr_start_end	#_of_RNA-seq_supporting reads
m84104_241218_092618_s2/95030008/ccs/8104_11348	TADA3	NC_051784.1	10055389-10057347	WDR5	NC_051789.1	6407509-6418207	23
m84104_241218_092618_s2/161481632/ccs/1486_3404	NUP153	NC_051773.1	111032094-111034826	LOC113842094	NC_051773.1	98695390-98698973	329
m84104_241218_092618_s2/162268296/ccs/15197_16784	LOC119714871	NW_024009727.1	197771-200762	LOC113841660	NC_051772.1	123599071-123600704	61
m84104_241218_092618_s2/112526548/ccs/4487_8193	LOC101800461	NC_051791.1	400-36792	GARNL3	NC_051789.1	12043197-12060077	7
m84104_241218_092618_s2	PICK1	NC_051772.1	54035920-54043570	TMCO1	NC_051779.1	10491132-10501066	518

/1534863<br>02/ccs/26<br>77_4570			

Note: Only representative fusions are shown here. The full table is provided in Supplementary File 2 (Excel format). RNA-seq supporting reads indicate the number of Illumina reads supporting the fusion junction.

Table S8. A list of the top 20 significantly upregulated and downregulated DEGs

Gene name	Log2FC	Pvalue	Regulation
NPY	-5.397021367	1.13227477752303e-15	down
H4	-11.25480482	1.53998487978754e-15	down
ABCB3	10.32450699	2.14824761018979e-13	up
PPP5C	8.14163494	3.00771618500748e-10	up
UBE2N	9.173789056	4.22850759935663e-10	up
TOM5	-5.468615462	2.21966044622877e-09	down
H3	-8.312080672	5.70421202756957e-09	down
MEF2A	22.40931125	9.78442321010019e-09	up
ERV3-1	22.33315387	1.09836931151665e-08	up
CHST1	-22.31976248	1.12178354097967e-08	down
CFAP46	-21.94819209	1.95645621706009e-08	down
ABCB2	8.468110148	2.96885460026825e-08	up
H3	-7.93386876	3.0059434325465e-08	down
LAMA4	4.304279736	4.20532205680439e-08	up
CMPK1	21.09200821	6.85896243795535e-08	up
TRDN	-5.957125678	9.39006321752903e-08	down
HHAT	5.831856519	1.03948259390711e-07	up
SLC25A17	4.279899662	1.18785413897158e-07	up

WBP4	5.738447501	2.47627186538826e-07	up
PCBD1	-5.176542087	3.21365703788784e-07	down

Table S9.Primer sequences for qPCR.

**Target gene**	**GenBank accession**	**Primer pairs(5'→3')**	**Product size(bp)**
H3	XM_038166278.2	F：GATCGCGCAGGACTTCAAGA<br>R：TCCTTGGGCATGATGGTGAC	150
CENPA	XM_038187986	F：TATCAGAGCACCACCAACCTG<br>R：CATGGCTTGCCATTGGTAGTC	108
ECM29	XM_038170014.2	F：CAGCATGGACTCGGATCAACT<br>R：TACGCACTCCTTCCTGTGTG	140
UPK1B	XM_027449623.3	F：GGTCATTGGGATGTGCGGTA<br>R：CCAGGCAGCAGCATAGATGT	121

Abbreviations: F, forward primer. R, reversed primer.

Table S10. Gene list overlapping with 3’UTR

3′ UTR lengthening / shortening	Gene
shortening	ETNK1、MXRA7、MCAM、VEGFC、HMGCS1、GCHFR、NOL12、ENC1、ZFYVE27、POSTN、RAB26、HNRNPD、NAT9、NRN1、DHCR24、UBALD1、EIF4EBP1、CLNS1A、EEFSEC、RNF19B、GCH1、WSB2、ST13、SCD、PSAT1、SPON1、HRAS、THY1、EDF1、DDIT4、SWI5、NR5A1、ALDOC、PPDPFL、HS3ST3B1
lengthening	SOD1、EXOSC6、PGR、KLHDC8A、SELENOH、RTN1、ALDH1A2、POLR2F、

	UPF3B、PEBP1、HOMER3、ANAPC11、FAM120B、STMN1、PTPT4A1

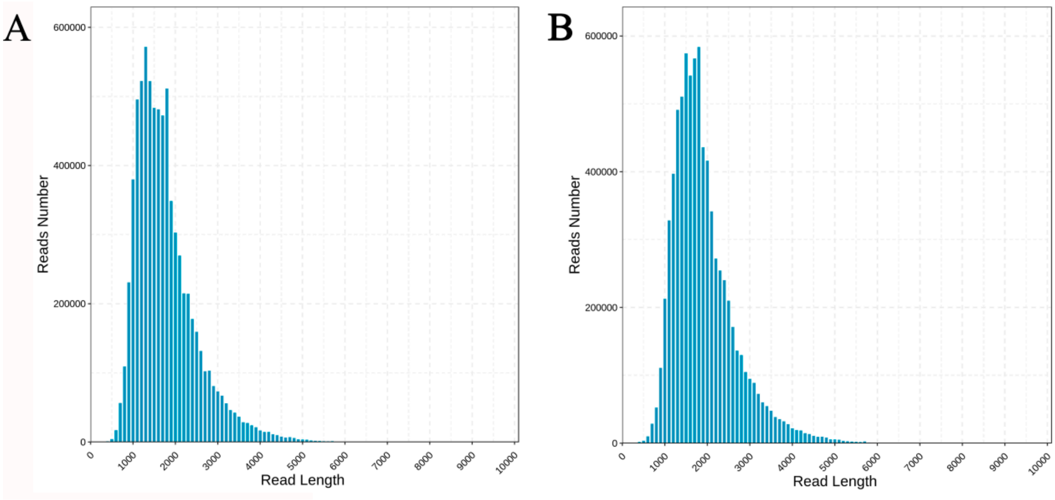

Figure S1. The predictive analysis of novel transcript. (A) The distribution of CCS lengths of QD. (B) The distribution of CCS lengths of SD. QD: Qiangying Duck, meat-type; SD: Shaoxing Duck, laying-type.

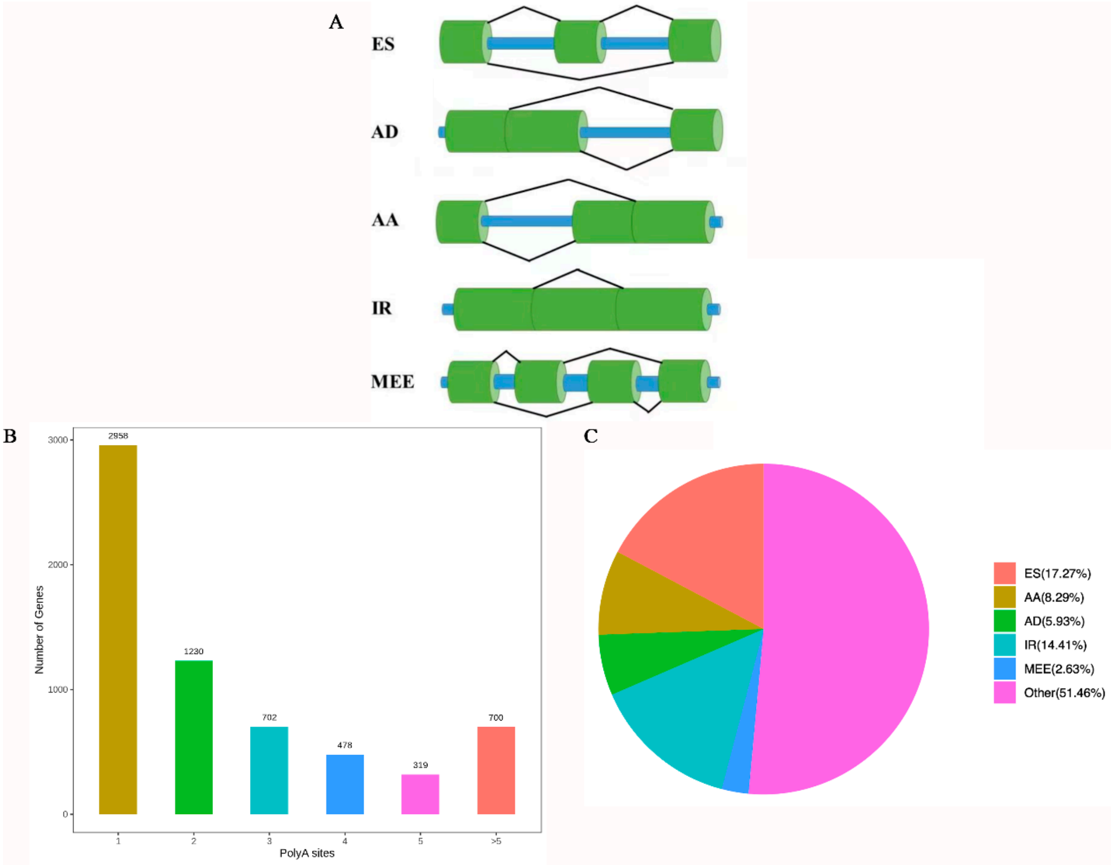

Figure S2. Analysis of AS events. (A) Classification diagram of AS events. (B) A bar chart with a variable number of AS event types. (C) Pie chart of the proportion of each type of AS event.
